# Plant extinction excels plant speciation in the Anthropocene

**DOI:** 10.1186/s12870-020-02646-3

**Published:** 2020-09-16

**Authors:** Jian-Guo Gao, Hui Liu, Ning Wang, Jing Yang, Xiao-Ling Zhang

**Affiliations:** 1 Department of Ecology, College of Urban and Environmental Sciences, and Key Laboratory for Earth Surface Processes of the Ministry of Education, Peking University, No.5 Yiheyuan Road Haidian District, Beijing, 100871 China; 2 Key Laboratory of Vegetation Restoration and Management of Degraded Ecosystems, Chinese Academy of Sciences, 723 Xingke Road, Guangzhou, 510650 China; 3 State Key Laboratory of Plant Cell and Chromosome Engineering, Institute of Genetics and Developmental Biology, Chinese Academy of Sciences, Beijing, 100101 China; 4 State Key Laboratory of Genetic Engineering, Collaborative Innovation Center of Genetics and Development, Department of Biostatistics and Computational Biology, School of Life Sciences, Fudan University, Shanghai, China; 5 Department of Public Policy, City University of Hong Kong, Tat Chee Avenue, Kowloon, Hong Kong, China

**Keywords:** Anthropocene, Biodiversity, Conservation, Plant extinction, Plant speciation

## Abstract

**Background:**

In the past several millenniums, we have domesticated several crop species that are crucial for human civilization, which is a symbol of significant human influence on plant evolution. A pressing question to address is if plant diversity will increase or decrease in this warming world since contradictory pieces of evidence exit of accelerating plant speciation and plant extinction in the Anthropocene.

**Results:**

Comparison may be made of the Anthropocene with the past geological times characterised by a warming climate, e.g., the Palaeocene-Eocene Thermal Maximum (PETM) 55.8 million years ago (Mya)—a period of “crocodiles in the Arctic”, during which plants saw accelerated speciation through autopolyploid speciation. Three accelerators of plant speciation were reasonably identified in the Anthropocene, including cities, polar regions and botanical gardens where new plant species might be accelerating formed through autopolyploid speciation and hybridization.

**Conclusions:**

However, this kind of positive effect of climate warming on new plant species formation would be thoroughly offset by direct and indirect intensive human exploitation and human disturbances that cause habitat loss, deforestation, land use change, climate change, and pollution, thus leading to higher extinction risk than speciation in the Anthropocene. At last, four research directions are proposed to deepen our understanding of how plant traits affect speciation and extinction, why we need to make good use of polar regions to study the mechanisms of dispersion and invasion, how to maximize the conservation of plant genetics, species, and diverse landscapes and ecosystems and a holistic perspective on plant speciation and extinction is needed to integrate spatiotemporally.

## Background

Today, humans are the dominant animal species on Earth, and we have both a direct (dramatically changing land surfaces by settlements) [1, 2] and indirect influence on the Earth’s climate [3–5], consequently changing the physiology, behaviour, and evolution trajectories of all other organisms [6–9]. The impact of climate warming is so strong that it influences almost everything from microorganisms to plant and animal populations. Climate change accelerates plant extinction by changing their phenology, e.g., mismatching the flowering period of plants with pollinating time of insects [10, 11] and narrowing the range of physiological adaptation, thus reducing plant resistance to extreme weather events [12, 13]. These effects are particularly substantial in hotspot areas of plant diversity in the tropics and subtropics [14–19]. On the other hand, climate warming has led to increased global vegetation activity, providing more resources and better hydrothermal conditions which are necessary for the evolution of new species [8, 20–24]. Recently, it has been observed that the plant diversity at a mountaintop increased significantly under the influence of climate warming [25], and studies have also showed that plant diversity continued to increase at high latitudes [26], leading to the conclusion that the Anthropocene is and will continue to be the golden age for the evolution of new species. The accelerated plant speciation in natural and unnatural ecosystems during this or the following centuries [23, 27–30] means that we will witness a world of diverse plants thriving in new environmental conditions. The two abovementioned viewpoints propose contrasting scenarios, i.e., the first indicates that plant speciation is accelerating in the Anthropocene, whereas the second indicates that plant extinction is simultaneously accelerating. The question that is important to address is if plant diversity will increase or decrease in this and following centuries [9].

Considering the development and utilization of plant resources, during the last 10,000 years, we have gradually acquired immense knowledge of plant species. Since the end of the Last Glacial Maximum, the Earth’s climate has warmed. The establishment of human settlements led to the development of agriculture and consequently, the development of modern civilization. Agriculture is a symbol of human civilization [31] and it is inseparable from plant domestication. Today, steamed bread made from hexaploid wheat, triploid banana, and seedless watermelons are consumed daily throughout the world, oils are extracted from tetraploid peanuts and clothing is made from tetraploid cotton. Domesticated plants, which some of them are polyploids, have higher leaf nitrogen content, higher growth rate and bigger seeds than those of their diploid ancestors, as polyploids with larger genome sizes are more resistant to adverse effects of genetic mutations [32, 33]. Today, the cultivation and management of polyploid crops support a population of 7.7 billion. Polyploid species generally evolve as a consequence of doubling of the chromosomes of the ancestral diploid. In recent times, the technology of inducing neopolyploidy by physical and chemical agents has been developed and is used in research. However, the technology used today for developing new polyploids is essentially the same as polyploidization in nature, except that the breeding cycle is greatly shortened using novel methods [34]. Compared with the human technology for developing neopolyploids, in nature, new species often evolve because of climate or environmental changes [29, 34–36].

### The dominant plant speciation type in nature

**Fig. 1:**
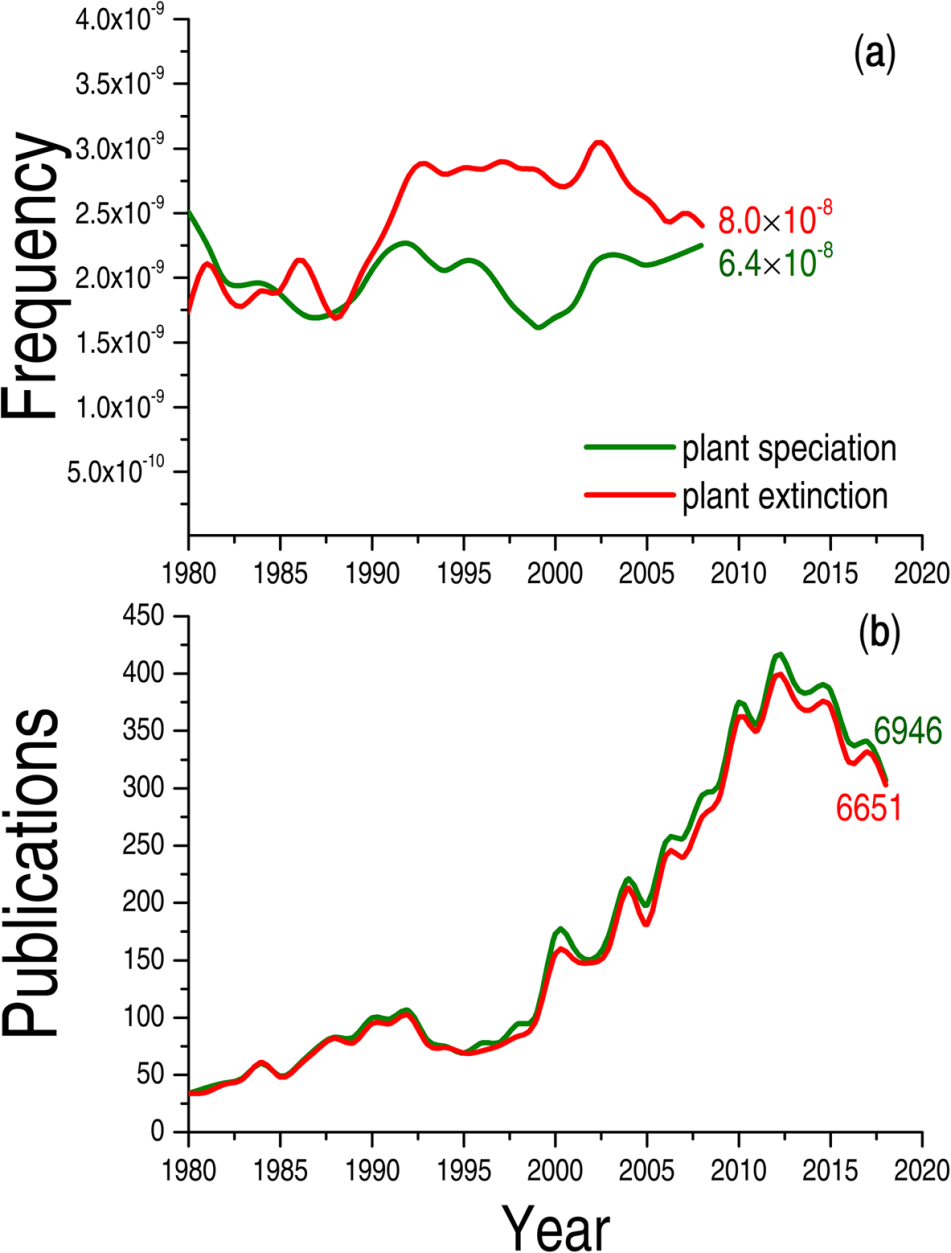
Differences of public and scientific attention to plant speciation and extinction. The public is more concerned with plant extinction (**a**), whereas scientists have a greater interest in plant speciation (**b**). We used Google Ngram’s millions of English-language books [37] to quantify the public attention on “plant speciation” and “plant extinction” during 1980–2008. The cumulative word frequency is 6.4 × 10^− 8^ and 8.0 × 10^− 8^ for plant speciation and extinction, respectively. We searched the core database of Web of Science for the publication of “plant speciation” and “plant extinction” during 1980–2018 to illustrate the academic concerns. The cumulative published papers are 6946 and 6651 on “plant speciation” and “plant extinction”, respectively. The searched research field was plant science, and the selected 10 journals of evolution and ecology: *Annual Review of Ecology Evolution and Systematics*, *Biology Letters*, *BMC Evolutionary Biology*, *Evolution*, *Evolutionary Applications*, *Evolutionary Biology*, *Evolutionary Ecology*, *Proceedings of the Royal Society B-Biological Sciences*, *Systematic Botany*, and *Trends in Ecology & Evolution*

**Fig. 2:**
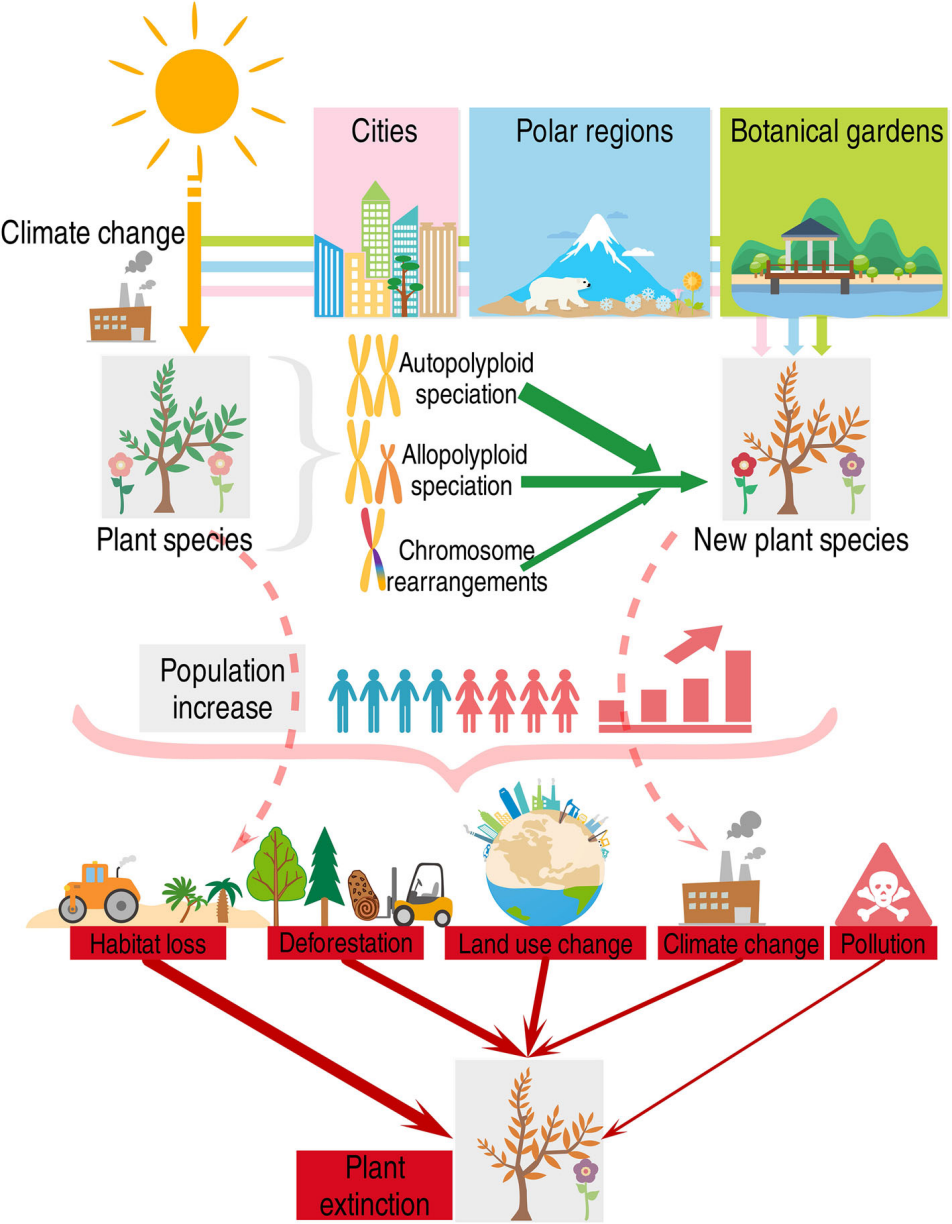
A simplified conceptual model depicting the types of plant speciation and the drivers of plant extinction in the Anthropocene. Three identified plant speciation accelerators, i.e., cities, polar regions, and botanical gardens are illustrated to show how climate warming might change plant evolution in the future. The contribution to plant speciation displayed from the top to bottom are: autopolyploid speciation, allopolyploid speciation, and chromosomal rearrangements. The main drivers of plant extinction displayed from right to left are: habitat loss, deforestation, land use change, climate change, and pollution. The solid lines denote the biological and ecological processes of plant speciation and extinction, in which the green ones denote the corresponding speciation types, and the red ones denote the five drivers of plant extinction. The thickness of the arrow denotes the relative strength of the contributions. It should be noted that climate change accelerates plant speciation while drives plant extinction either, and human population increase as the primary driver of plant extinction. Both new plant species and their progenitors of plant species may face same extinction risk in the Anthropocene, but the new plant species are more likely to survive due to their stronger natural adaptability to climate change [38]

Even though humans have been breeding new plant species for the past few thousand years, more focus has been placed on plant extinction than on plant speciation (Fig. 1) [37]. With the acceleration of climate warming, plant speciation caused by warming climate may become a more common phenomenon in this and following centuries [27]. Human civilization supports itself by intensive exploitation and utilisation of fossil energy accumulated approximately 300 million years ago (Mya). The CO_2_ emissions from a large number of fossil combustions are the main factor contributing to warmer climate and associated issues, such as ocean acidification and species extinction. Species extinction and conservation are important matters of public concern (Fig. S1). Comparing the extinction risk of species, the formation of new plant species is thought to be currently constrained in the academic world [27], this is partly because people (non-scientists) become aware of species extinction reports (Fig. 1; Fig. S1). However, plant speciation through polyploidization may be quite common, and autopolyploidy, which is caused by the polyploidization of two conspecific individuals, is presumed to be the dominate [27, 28, 34] (Fig. 2). On the other hand, allopolyploids are the result of interspecific hybridization process and chromosome duplication. Speciation as a consequence of chromosomal rearrangements, homoploid hybrid speciation, and lineage splitting are not common because of their long evolutionary time and low occurrences, e.g., it takes several thousand years for a new plant species to evolve through lineage splitting [39].

### Speciation accelerated by the greenhouse effect

Understanding that autopolyploid speciation is the most dominant speciation type in the Anthropocene requires a view from the perspective of plant evolution. The Earth has experienced extensive climatic fluctuations since its birth. The first plants are nearly omnipresent on Earth and they often survive climatic fluctuations via whole genome duplication, i.e., polyploidization [35]. For example, many species of ferns, gymnosperms, and angiosperms have undergone multiple whole genome duplications during their long evolution [40]. The main biological determinant of plant survival is their genome multiplication. For example, during the warm period of the Palaeocene–Eocene Thermal Maximum (PETM) 55.8 Mya, when the Earth experienced temperatures 5–8 °C higher than those today, the multiplication of the genomes of many angiosperm species occurred [38, 41, 42]. Multiplications provided angiosperms the advantage in surviving sudden climate changes by giving them a competitive growth advantage, and they were eventually established as the most successful plant lineage [36, 40, 43–47]. While climate warming could disturb germline development and lead to reduced fitness in some plant species [48], plants could also evolve to adapt because the production of unreduced gametes that facilitates autopolyploidization is positively correlated with nutrient (e.g., nitrogen deposition and crops fertilization), humidity (e.g., floods and reduced surface runoff due to elevated CO_2_), temperature variation (e.g., heat waves) and high levels of herbivory (i.e., bark beetle and caterpillars outbreaks) that likely to be encountered under future climate change scenarios [27, 34]. Considering that the climate at the end of this century could resemble that during PETM [49], and if greenhouse gas emissions are not significantly reduced, it is reasonable to infer that plants will experience polyploidy of a similar magnitude.

### Mechanisms underlying plant speciation

Review the past to understand the present. According to the Representative Concentration Pathway 8.5 (RCP 8.5) emission scenario, climate warming today might promote polyploidy to a similar extent to that during PETM. The question is, what are the biological or ecological mechanisms underlying the increase in polyploidization? There are two possible reasons for this increase [34, 48]: (i) the first is high temperature induction of chromosome doubling. In a newly fertilized immature embryo or germ cell, active division occurs, meaning it is easy to induce polyploidy by sudden temperature increases (but not as high as to cause cell death). Autotetraploid corn was developed using this method prior to the discovery and extensive use of colchicine; (ii) Climate warming leads to the extension of the growing season. This increases the chances of contacts among plants, and thus sympatric speciation emerges as the dominant speciation type [50]. Significant results of climate warming are the advance of budding and the delay in leaf shedding, both of which prolong the growing season [51]. These scenarios favour the breakage of prezygotic barriers. In the foreseeable future, climate warming will be the dominant climatic feature of this and the following centuries [52], thereby ensuring the occurrence of autopolyploid speciation [35].

### Accelerators of plant speciation

Plant resources determine the sustainable development of human society, and thus, nearly all countries have made great efforts to establish protected areas to maintain their plant diversity [53, 54]. A large number of protected areas worldwide have significantly reduced habitat loss [55–57], and large unexplored wilderness areas have reduced the risk of plant extinction by at least 50% [58]. Compared to larger and more effective protected areas (~ 15%) [53] or wilderness (~ 23%) [59], urban development areas account for only ~ 1% of land surface area [60], but they have extremely significant effects on plant phenology, physiology, ecology, and heredity [61–63]. In addition, the genetics of the plant species in botanical gardens are also significantly different from their wild congeners [64]. In the Anthropocene, the three accelerators that have a significant effect on driving plant species evolution are cities, the polar amplification of climate change, and botanical gardens which are believed to play both the role of “cradle” and “museum”.

#### Accelerator I: cities

One of the most prominent features of the Anthropocene are man-made clusters of large buildings and cities. As a “natural laboratory”, the city is an ideal environment for monitoring the rapid evolution of plants [65]. Cities are inhabited by more than 50% of the world’s population and consume ~ 80% of global energy [60]. As a consequence of the pronounced “heat island effect”, it has been predicted that urban development will become an accelerator of plant speciation. The temperatures of most cities are ~ 1 °C higher than those of the surrounding non-urban areas, and the temperatures in humid regions or densely populated cities can be even 1.5 °C higher than those of the surrounding non-urban areas [66]. Therefore, the climate of cities today is comparable to the predicted climate at the end of the century (equivalent to significant carbon mitigation in the RCP 2.6 scenario). As most urban plant species are native species with strong adaptability [67, 68], and most importantly, because these native urban plants are planted and grown only in extremely limited space, these plants are more likely to undergo autopolyploid speciation than any other speciation type [65].

#### Accelerator II: polar regions

As a consequence of polar amplification [69], the Arctic may be the fastest warming region on Earth compared to the global average (0.60 vs. 0.17 °C/decade) [70]. Melting ice further reduces solar radiation reflections, which further accelerates the melting of glaciers, eventually leading to an ice-free summer predicted for the eve of 2030 [71–73]. The warm Arctic waters are favourable for phytoplankton [74], and the increase in phytoplankton significantly increases the primary producers’ provision for marine animals, which in turn promotes the prosperity of biodiversity [75]. Polar regions (including Antarctica and the Qinghai-Tibet Plateau) have always been the centre of plant divergence [76, 77], and they may play more of a “cradle” role in the context of climate warming. It has recently been found that the number of plant species has significantly increased in the Arctic [26], which might have been a consequence of diffusion or migration [78] rather than speciation, suggesting a “museum” role of the polar regions. With the further warming of the global climate, especially in the Antarctic [79, 80] and the “Third Pole”, i.e., Qinghai-Tibet Plateau [81] that have similar temperature increase amplitudes to the Artic, new species may evolve from alien and native species through hybridization or autopolyploid speciation in these polar regions.

#### Accelerator III: botanical gardens

Early plant gatherers collected exotic plants and planted them in small areas; today, these areas would be called botanical gardens. Botanical gardens are spread worldwide because of their practical functions, such as cultural and recreational function as well as medicinal plant preservation [82]. When plant gatherer Ernest Henry Wilson brought thousands of plant species from China and Asia to Kew Botanic Garden, he could not have known that his actions would inadvertently lead to the evolution of a new species, *Primula kewensis* [23], as well as many other new plant species (cf. Chris D. Thomas’s papers). There is a possibility that some epiphytic orchids in the botanical garden are currently undergoing rapid speciation [64]. The attraction of people to ornamental flowers has led to the development of new species of orchids and primroses [83, 84], indicating that botanical gardens can be used as cradles of speciation. The main reason why botanical gardens are a major factor in the evolution of new species is that a large number of plants gathered in a small area increases the probability of interspecific pollen transmission via pollinating insects. Therefore, in addition to autopolyploid speciation, allopolyploidy and hybrid speciation are also important in plant evolution in these artificial gardens [23, 24, 29, 85–88]. Botanical gardens preserve ~ 30% of plant species and ~ 40% of endangered plants on the Earth, and thus they have an extremely important conservation function [89]. One of the most obvious features is that the plants found in botanical gardens may have come from any corner of the world [90]. Therefore, the contribution of allopatric and that of sympatric speciation to the evolution of new species may be equally important.

### Bigger extinction

#### The alarming risk of extinction

Polyploidy promotes genetic diversity, which is why it is a common plant strategy for surviving climate change [27]. The accelerated speciation under climate warming conditions enhances their adaptation and resilience to climate change, which is necessary to maintain plant biodiversity and crucial for survival. For example, fossil evidence suggests that plants were resilient to mass extinction [91], and had fewer extinctions compared to marine fauna. Under the climate warming scenario, the resulting accelerated plant speciation could be 50–300 times faster than background speciation, and this rate is far lower than the current rate of plant extinction. In contrast, the rate of plant extinction in the Anthropocene is 1000–10,000 times higher compared to that of the background extinction [27, 92]. Therefore, as a consequence of mankind’s inconsiderate exploitation of the Earth’s resources, as well as land-use changes, habitat loss and international trade [9, 93], the risk of plant extinction is much higher than the possibility of plant speciation (Fig. 2). For example, Marques et al. (2019) estimated that 33% of Central and Southern America and 26% of Africa’s biodiversity were influenced by consumption in other world regions [93].

#### The worsening tropics

Although the risk of plant extinction in Europe (non-tropical areas) is not high, and regional plant diversity may be increasing, the abundance of plant species in the tropical regions with highest species richness (i.e., Congo, Amazon, and Southeast Asia) [94] is decreasing and that’s why the risk of global overall plant extinction still appears to be very high [15, 16, 95]. For example, 58% of tree species in Amazonia are predicted to go extinct in the following 30 years under the pressures of deforestation and climate change [94].

#### Trophic cascading

The indirect effects of anthropogenic activities may be more devastating to plants than we perceive. The annual decline in insect biomass is estimated to be 2.5% worldwide [96]. As insects are the basis of terrestrial and aquatic food chains, the increase in prezygotic barriers would be devastating for plant diversity [87]. For example, in some very small populations of endangered plants, the decrease in the number of pollinating insects can increase the pollen limit, thus increasing the risk of extinction [97, 98]. The decrease in the number of pollinating insects negatively affects not only endangered plants, but almost all plants that are pollinated by insects, especially certain crops crucial for agriculture [99, 100]. In recent years, large-scale use of chemicals such as neonicotinoid pesticides has caused irreversible damage to bee populations [101–103]. Even worse, the dramatic decline in bird populations is similar to the dramatic decline in insect populations. For instance, a recent study showed that North American avifauna has decreased by 3 billion over the past half century, which is equivalent to ~ 30% of the total number of birds in the 1970s [104]. Today, the sharp decline in bird populations as a consequence of habitat loss because of agricultural activities, urbanization, and toxic pesticide use in both breeding and wintering areas is a global problem [105, 106]. Pollinating insects and birds are directly related to plant life histories (e.g., reproduction and seed dispersal), which is why their continuous and significant decrease in numbers will have a significant negative impact on plant diversity [99, 107–109].

### Humans determine plants extinction directly and globally

In the next ten years, the global population is predicted to reach 8.5 billion [110] and surge to 9.7 billion and 11 billion in the middle and the end of this century, respectively [111]. Such a large population will have significant impacts on the Earth’s resources and natural ecosystems [112, 113]. In order to feed such a large population, 100–110% increase in global crop supply must be achieved by the middle of this century, which means that without the implementation of high-efficient agriculture, arable land area will have to increase by approximately 1 billion ha [114]. In order to achieve this, deforestation will be unavoidable [115, 116], which will cause a large number of forests to be converted into agricultural land and settlements [31, 117], and these land-use conversions will lead directly to plant habitat loss, which is the direct reason of plant extinction [18] (Fig. 2). Therefore, deforestation and land use change can be considered as the main and direct causes of plant extinction, whereas climate change and pollution are indirect (or possibly partly direct) causes of plant extinction [118]. It has been predicted that heavy metal and synthetic chemical pollution will change pollen morphology and physiological functions of plants, leading to the extinction of terrestrial plant species [119, 120], and excessive use of nitrogen and phosphorus fertilizers in agriculture leads to eutrophication and extinction of both terrestrial and aquatic plant species [121, 122].

In addition, fire [123] or outbreaks of pests [124] and invasive plants [125], which can be indirect effects of climate change, will also significantly increase the risk of plant extinction. The driving forces of plant extinction are not mutually exclusive [14, 126]. For example, land use change is the result of deforestation, which can further enhance the effects of climate change because the deforested areas are more negatively influenced by unstable climate extremes than forested areas. Deforestation and the corresponding habitat loss are direct causes of plant extinction [126], but by the middle of this century, their negative impacts could be surpassed by the negative impacts of climate change [94]. But here, climate change, mainly means a warming climate, is supposed to have a more significant effect on plant speciation [24, 127]. However, the positive effect of climate change on plant speciation was greatly reduced and reversed by land use change, deforestation, and habitat loss. Therefore, in the Anthropocene, the rate of plant speciation is much lower than the rate of plant extinction [128]. In all, the primary cause of plant extinction is the uncontrolled exploitation of the Earth’s resources in order to maintain human population growth and quality of life [93, 129, 130].

## Conclusions and future perspectives

Sustainable development of the human society is closely related to plant diversity [131]. Plant diversity directly determines wood production [132] and plants/vegetation resistance to pests and pathogens inherently [133]. In addition, plant diversity is also closely related to food security [134], diet nutrition [135], and human health and disease transmission [136, 137]. Therefore, maintaining plant diversity essentially means maintaining the sustainability of human society [16, 138]. At present, only a few plant species are widely used as crops, and most plant species have great potential for unusual use [139], but we currently know very little about their status in the wild, especially for the plants inhabiting highest plant diversity areas, such as lowland tropics in Southeast Asia and the Amazon [14, 92]. As a consequence of the rapid increase in human population, habitat loss is almost inevitable [140], which could lead to plant extinction that would be no less in extent than the five major extinctions that occurred during the past, and this new extinction has already been termed as the “sixth mass extinction” [141–143]. As a consequence of the burning of large amounts of fossil fuels in a very short time, CO_2_ emissions and radiative force may exceed the highest levels in the past hundreds of millions of years [144]. It is difficult to conclude whether plants can quickly adapt to these environmental changes, but one thing is certain: because of the predatory exploitation of plant resources by human beings, plant diversity is unlikely to recover from this artificial mass extinction as well they did the past five times nor will the plants have the opportunity to undergo rapid speciation or evolve new plant species to fill ecological niches [145] as long as they are occupied by humans or crops [129, 146].

According to the simple principle of biodiversity-energy, it is inspired by a geological period, such as PETM of great biodiversity [8]. During this period, the climate is warm and humid, which is characterized by “crocodiles in the Arctic”. The diversity of animals and plants was very high during PETM, and the angiosperms have undergone whole genome duplications, allowing them to dominate the terrestrial flora [40–43, 147, 148]. As the temperature of RCP 8.5 characterized by high emissions has many similarities with PETM [49], we speculate that climate warming in the twenty-first century will accelerate plant speciation via autopolyploidization [27, 28]. Although plant species turnover will be faster in this century than in the previous centuries, the rate of plant extinction caused directly by land use change and deforestation will be much higher than the rate of plant speciation accelerated by climate warming. Therefore, the risk of plant extinction in the Anthropocene is still very high.

We suggest several topics for future research: (i) Because rapid climate change and land use change will inevitably lead to the rapid evolution of some plants, it is important to study which plant traits are more likely to change, how they affect speciation and extinction, and what are their implications for conservation and plant adaptation [106, 149]. Implementing this kind of research in cities and botanical gardens may be the best choice; (ii) Polar regions face great direct and indirect pressures from the consequences of climate change and human exploitation. They are the outposts in response to climate change and we need to make good use of these natural laboratories to research the mechanisms of dispersion and invasion which cause plant speciation and extinction; (iii) It is also important to study how to maximize the conservation of plant genetics, species, and landscape and ecosystem diversity in the context of the development of human society and well-being [150]. Establishing a framework of “nature-social-health nexus” which incorporates the resilience of ecosystems, optimizes the efficiency of the framework by practice, and maximizes the sustainability of the human society is extremely important [108, 114]; (iv) The aim of these researches would be to gain a holistic perspective on plant speciation and extinction. As today, speciation has been more researched from genetic or molecular perspectives, whereas extinction has been researched from ecological perspectives, we conclude that both views are needed to integrate plant evolution spatiotemporally.

## Supplementary information

**Additional file 1: Figure S1.** Relative search frequency of the terms “plant speciation” and “plant extinction” via Google (Google Trends). Time span from January 2004 to July 2019.

## Data Availability

The datasets in this study are available from the corresponding author on reasonable request.
