# Supplementary material for: Plant extinction excels plant speciation in the Anthropocene
Source: BMC Plant Biol. 2020 Sep 16;20:430. doi: 10.1186/s12870-020-02646-3 (PMC7493330; doi:10.1186/s12870-020-02646-3)
Supplement: Supplementary file 1 — Additional file 1: Figure S1. Relative search frequency of the terms “plant speciation” and “plant extinction” via Google (Google Trends). Time span from January 2004 to July 2019. [file 12870_2020_2646_MOESM1_ESM.pdf]

Supplementary information

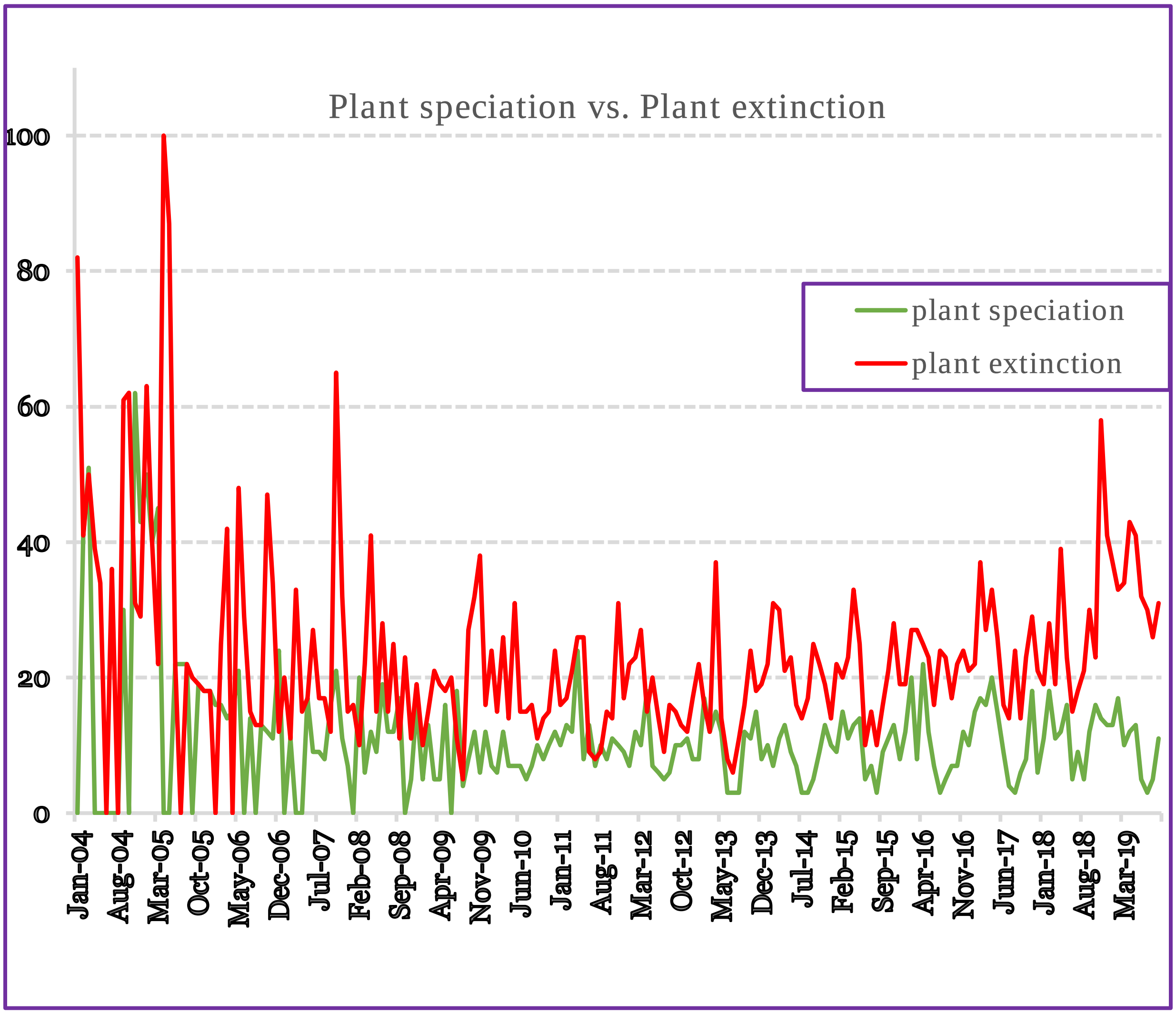

Fig S1. Relative search frequency of the terms “plant speciation” and “plant extinction” via Google (Google Trends). Time span from January 2004 to July 2019.
